# NLRP3 inflammasome has a protective effect against oxazolone-induced colitis: a possible role in ulcerative colitis

**DOI:** 10.1038/srep39075

**Published:** 2016-12-14

**Authors:** Shigehiro Itani, Toshio Watanabe, Yuji Nadatani, Naoki Sugimura, Sunao Shimada, Shogo Takeda, Koji Otani, Shuhei Hosomi, Yasuaki Nagami, Fumio Tanaka, Noriko Kamata, Hirokazu Yamagami, Tetsuya Tanigawa, Masatsugu Shiba, Kazunari Tominaga, Yasuhiro Fujiwara, Tetsuo Arakawa

**Affiliations:** 1 Department of Gastroenterology, Osaka City University Graduate School of Medicine, Osaka, Japan; 2 SAMURAI International GI Research Center, Osaka, Japan

## Abstract

The inflammasomes induce maturation of pro-interleukin-1β (IL-1β) and pro-IL-18. We investigated roles of the NLRP3 inflammasome in the pathogenesis of ulcerative colitis (UC). After induction of oxazolone-induced colitis, a mouse UC model, colonic tissues were assayed for inflammatory mediators. Histological studies were performed on inflamed colonic tissue from mice and UC patients. Histological severity of murine colitis peaked on day 1, accompanied by an increase in the expression of Th2 cytokines including IL-4 and IL-13. Oxazolone treatment stimulated maturation of pro-caspase-1 and pro-IL-1β, while it reduced IL-18 expression. Either exogenous IL-1β or IL-18 ameliorated the colitis with or without reduction in Th2 cytokine expression, respectively. Induction of colitis decreased MUC2 expression, which was reversed by administration of IL-18, but not IL-1β. Compared to wild-type mice, NLRP3^−/−^ mice exhibited higher sensitivity to oxazolone treatment with enhancement of Th2 cytokine expression and reduction of mature IL-1β and IL-18 production; this phenotype was rescued by exogenous IL-1β or IL-18. Immunofluorescent studies revealed positive correlation of NLRP3 expression with disease severity in UC patients, and localization of the inflammasome-associated molecules in macrophages. The NLRP3 inflammasome-derived IL-1β and IL-18 may play a protective role against UC through different mechanisms.

Ulcerative colitis (UC), which is a representative inflammatory bowel disease (IBD) together with Crohn’s disease (CD), is characterized by a T helper cell type (Th) 2 immune response with contiguous mucosal inflammation in the rectum and colon that cause epithelial barrier dysfunction and lead to ulceration^1^.

Many models of colitis have been developed to investigate the pathophysiology of IBDs. Among these models hapten-induced colitis, in which oxazolone (OXA: 4-ethoxymethylene-2-phenyl-2-oxazoline-5-one) is delivered intrarectally to rodents, is regarded as a model of UC. This model of colitis is driven by the production of Th2 cytokines, such as interleukin-4 (IL-4) and IL-13, and recapitulates the disease features of human UC in terms of histological findings, affected site of injury (i.e., rectum), and Th1/Th2 cytokine balance^2^,^3^.

Recent studies have shown that the maturation and secretion of IL-1β and IL-18 are managed by the inflammasome, an intracellular multiprotein complex^4^. Inflammasomes are comprised of a pattern recognition receptor (PRR) such as Nod-like receptor family pyrin domain-1 containing 3 (NLRP3), an adaptor protein, apoptosis-associated speck-like protein containing a caspase recruitment domain (ASC), and pro-caspase-1^5^. Recognition by the PRR of endogenous and exogenous signals arising from intracellular or extracellular stressors triggers the assembly of the inflammasome, leading to the caspase-1-dependent processing of pro-IL-1β and pro-IL-18, allowing for the secretion of the mature active forms of these cytokines^6^.

Recent genome-wide association studies have found that polymorphisms conferring a hypofunctional NLRP3 phenotype are associated with development of CD, suggesting a protective role for the NLRP3 inflammasome in the pathogenesis of CD^7^,^8^. However, there are few studies to assess the role of the NLRP3 inflammasome in the pathogenesis of UC.

Animal studies using dextran sulfate sodium (DSS)-induced colitis and 2,4,6- trinitrobenzene sulfonic acid solution (TNBS)-induced colitis demonstrated that NLRP3^−/−^ and caspase-1^−/−^ mice exhibited severe colitis compared to wild-type (WT) mice; this aggravation of colitis was due to a lack of IL-18, although the opposite effect of NLRP3 and caspase-1 against DSS-induced colitis was reported by another group^9^,^10^,^11^. However, there are no studies to investigate the role of the NLRP3 inflammasome in a Th2 cytokine-dominant colitis model resembling UC.

In this study, we investigated the role of the NLRP3 inflammasome in the development of UC using human UC operation resection specimens and an OXA-induced colitis model.

## Results

### UC disease severity was reflected in the expression levels of NLRP3 and colocalization of NLRP3 with cleaved caspase-1 in the human colon tissues

**Figure 1:**
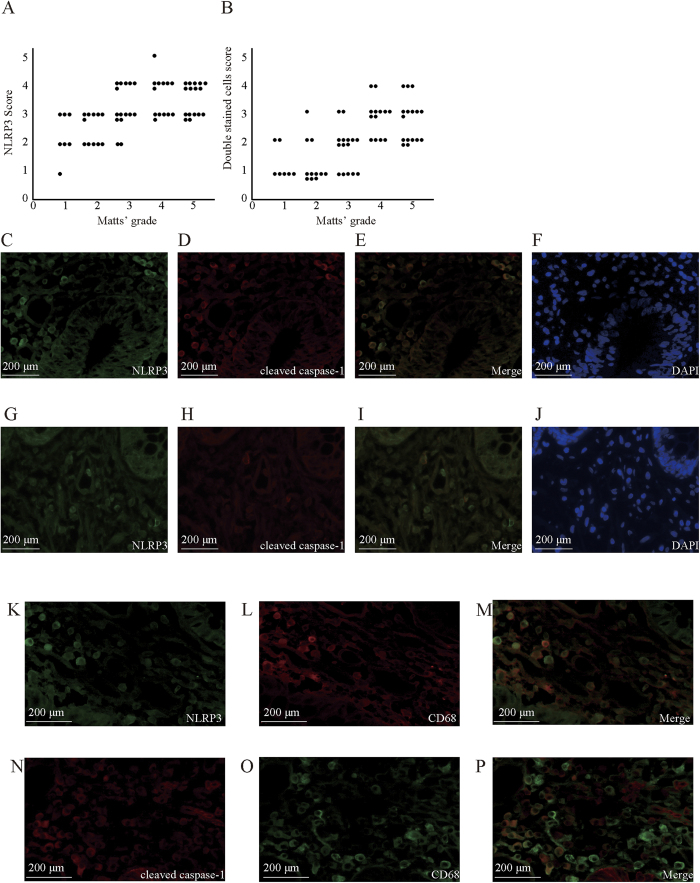
Histological and clinical evaluation in human colon operation resection specimens. (**A**) Correlation of the score of NLRP3 immunoreactivity and the Matts’ histopathological grading system score in the colonic mucosa of patients with ulcerative colitis (UC). The NLRP3 immunohistochemical score represents the average percentage of the NLRP3 positive cells relative to total lamina propria cells, which were counted in three microscopic fields at 400× magnification in each specimen. Matts’ score represents the histological severity of colonic specimens of UC patients (Supplementary Table 1). Each dot represents one colonic specimen of a UC patient. N = 62. (**B**) Correlation of the score on NLRP3 and cleaved caspase-1 double-stained cells and the score on Matts’ histopathological grading system in the colonic mucosa of UC patients. (**C**–**F**) Expression of NLRP3 (**C**), cleaved caspase-1 (**D**), double-staining of simultaneous expression of NLRP3 and cleaved caspase-1 (**E**), and DAPI staining (**F**) in the colon tissue samples from severe UC patients. The total numbers of NLRP3-positive cells, of double-stained cells, and all cells in the lamina propria were 64, 43, and 105, respectively. Thus 61% of the cells expressed NLRP3, and 41% of the cells were NLRP3 and cleaved caspase-1 double positive. (**G**–**J**) Expression of NLRP3 (**G**), cleaved caspase-1 (**H**), double-staining simultaneous expression of NLRP3 and cleaved caspase-1 (**I**), and DAPI staining (**J**) in the colon tissue samples from mild UC patients. The total numbers of NLRP3-positive cells, of double-stained cells, and all cells in the lamina propria were 16, 4, and 97, respectively. Thus, 16% of the cells expressed NLRP3, and 4.1% of the cells were NLRP3 and cleaved caspase-1 double positive. In UC patients, immunoreactivity for NLRP3 and cleaved caspase-1 was observed primarily in inflammatory cells as well as some epithelial cells (**C**–**J**). (**K**–**P**) Immunohistochemical double staining of NLRP3, caspase-1, and CD68, a marker of macrophages, in colonic tissue samples from UC patients. Double staining of NLRP3 (**K**–**M**) or cleaved caspase-1 (**N**–**P**) with CD68 demonstrated that the majority of these inflammatory cells were macrophages. The dosage and source of primary antibodies are listed in Supplementary Table 2.

The expression levels of NLRP3 in the colonic mucosa of patients with UC positively correlated with disease severity, as assessed by Matts’ histopathological grading system (Supplementary Table 1^12^; r = 0.57, p < 0.01, Fig. 1A). The protein levels of NLRP3, which was induced by intestinal inflammation, were markedly increased in the colon of patients with severe UC (Fig. 1C,G). The immunoreactivity for NLRP3 and cleaved caspase-1 was observed mainly in inflammatory cells and in some epithelial cells (Fig. 1C–F). The percentage of NLRP3 and cleaved caspase-1 double-stained cells, which reflected the NLRP3 inflammasome activation, also positively correlated with disease severity (r = 0.67, p < 0.01, Fig. 1B). Immunohistochemical double staining showed that the majority of NLRP3-positive cells strongly coexpressed cleaved caspase-1 in the colon of patients with severe UC (Fig. 1C–F), but a moderate number of double-stained cells was observed in the colon of patients with mild UC (Fig. 1G–J). Double staining of NLRP3 or cleaved caspase-1 with CD68 demonstrated that the majority of these inflammatory cells were macrophages (Fig. 1K–P).

### Intrarectal OXA administration caused UC-like colitis in mice

**Figure 2:**
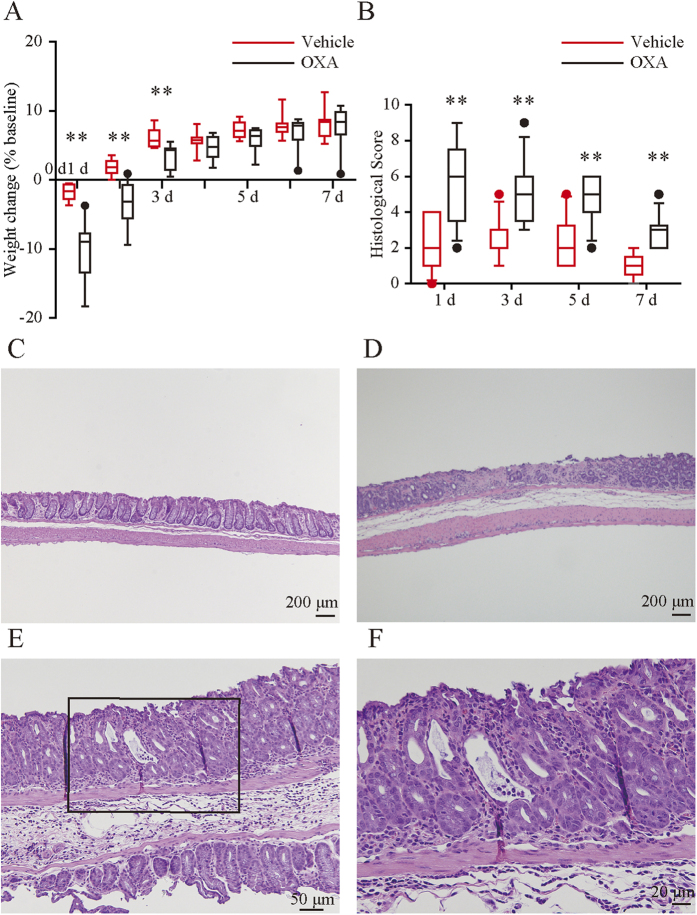
Changes in body weight and histological severity during the mouse oxazolone-induced colitis. (**A**) Changes in body weight during the experimental period. OXA, oxazolone. (**B**) Changes in histological score during experimental period. Each box plot represents median, 10–90% range, minimum, and maximum values. p values were calculated by post hoc steel significant difference multiple comparison. N = 9–14. **p < 0.01, *p < 0.05 compared with vehicle-treated mice. (**C**–**F**) Histological findings of colon samples in vehicle (**C**) or oxazolone-treated mice (**D**–**F**) on day 1. (**F**) Higher magnification view relative to the boxed area in (**E**). Oxazolone-treated mice revealed superficial inflammation characterized by epithelial cell loss (**D**), inflammatory cell infiltration composed mainly of neutrophils, and occasionally cryptitis (**E**,**F**), while the vehicle-treated mice exhibited very slight superficial damage (**C**).

The OXA-treated group developed rapid-onset colitis marked by weight loss and diarrhea. The median body weight increased significantly more for the mice treated with vehicle than for mice treated with OXA on days 1, 2, and 3 (Fig. 2A).

The histological score of the OXA-treated group peaked on day 1 and gradually declined. The score of the OXA-treated group was significantly higher than that of the vehicle group throughout the experimental period (Fig. 2B). Histological examination of colonic sections from the OXA-treated mice revealed superficial inflammation characterized by epithelial cell loss (Fig. 2D), inflammatory cell infiltration composed mainly of neutrophils, and occasionally cryptitis (Fig. 2E,F), while the vehicle-treated mice exhibited minor superficial damage (Fig. 2C).

### Expression of NLRP3 inflammasome-associated molecules and cytokines in the mouse colon was significantly increased by intrarectal administration of OXA

**Figure 3:**
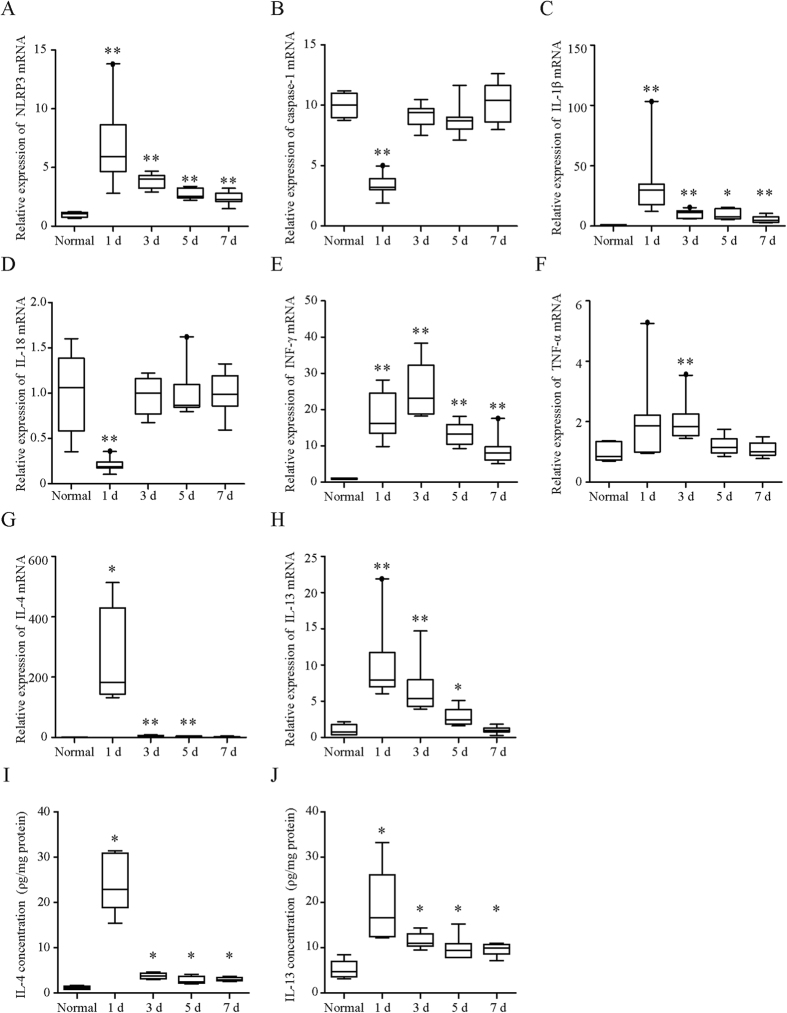
Changes in the NLRP3 inflammasome-associated molecules and cytokines during oxazolone-induced colitis. (**A**–**H**) mRNA levels of NLRP3 (**A**), caspase-1 (**B**), IL-1β (**C**), IL-18 (**D**), INF-γ (**E**), TNF-α (**F**), IL-4 (**G**), and IL-13 (**H**) in oxazolone-induced mice colonic tissue. The levels of indicated mRNA were determined by quantitative reverse transcription-polymerase chain reaction. mRNA levels are expressed as ratios, relative to the mean value for normal colonic tissue. (**I**,**J**) Protein concentration of IL-4 (**I**) and IL-13 (**J**) in oxazolone-induced mice colonic tissue. Protein concentrations were measured by the corresponding enzyme-linked immunosorbent assay. The protein levels in each sample were standardized using the modified bicinchoninic acid method. Each box plot represents median, 10–90% range, minimum, and maximum values. p values were calculated by post hoc steel significant difference multiple comparison. N = 9. **p < 0.01, *p < 0.05 compared with normal colonic tissue.

**Figure 4:**
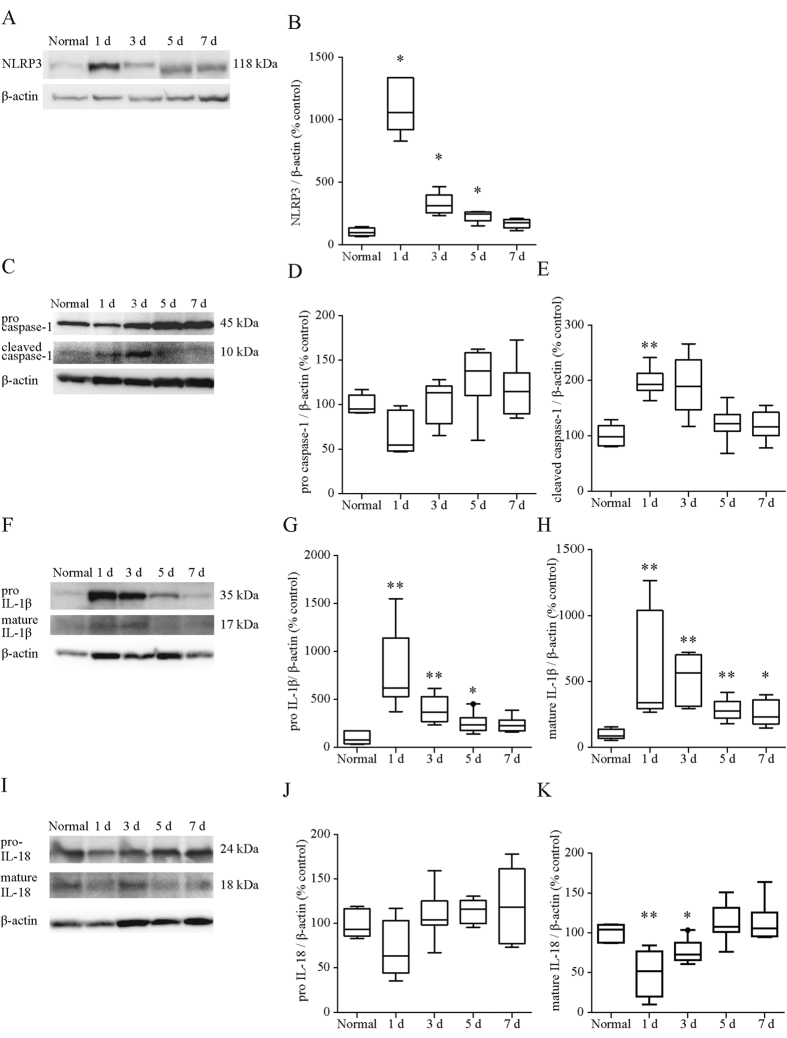
Protein expression of NLRP3 associated molecules after oxazolone treatment. Expression levels of NLRP3 (**A**,**B**), pro-(**C**,**D**) and cleaved caspase1 (**C**,**E**), pro-(**F**,**G**) and mature IL-1β (**F**,**H**), pro-(**I**, **J**) and mature IL-18 (**I**,**K**) were investigated by western blot using their corresponding antibodies (Supplementary Table 2) and relevant bands were quantified with laser-scanning densitometry. β-actin was used as the normalization control. Each box plot represents median, 10–90% range, minimum, and maximum values. p values were calculated by post hoc steel significant difference multiple comparison. N = 9. **p < 0.01, *p < 0.05 compared with normal colonic tissue. Protein levels are expressed as ratios, relative to the mean value for normal colonic tissue.

Expression of NLRP3 at both the mRNA (Fig. 3A) and protein levels (Fig. 4A,B) in the OXA-treated colon reached a peak on day 1 and then decreased with time. The expression levels of caspase-1 mRNA were significantly decreased by OXA-treatment on day 1 and returned to normal levels on day 3 (Fig. 3B). The protein levels of pro- caspase-1 were also non-significantly reduced on day 1 (p = 0.11, Fig. 4C,D), whereas those of cleaved caspase-1 were significantly elevated on day 1 (Fig. 4C,E). This significant elevation of cleaved caspase-1 in the OXA-treated group was no longer apparent on day 3.

Expression of IL-1β mRNA (Fig. 3C) and mature IL-1β protein levels (Fig. 4F,G,H) were markedly increased and reached a maximum on day 1 of OXA-treatment. On the other hand, expression of IL-18 mRNA (Fig. 3D) and mature IL-18 protein levels (Fig. 4I,J,K) were significantly reduced on day 1 but returned to normal levels thereafter. The OXA-treatment results in a prominent increase in both the levels of mRNA and protein concentration of IL-4 (Fig. 3G,I), and it induced modest increases in mRNA expression of Th1 cytokines (i.e., IFN-γ and TNF-α) as well as IL-13 (Fig. 3E,F,H,J).

### Exogenous IL-1β and 18 ameliorated OXA-induced colitis in mice

**Figure 5:**
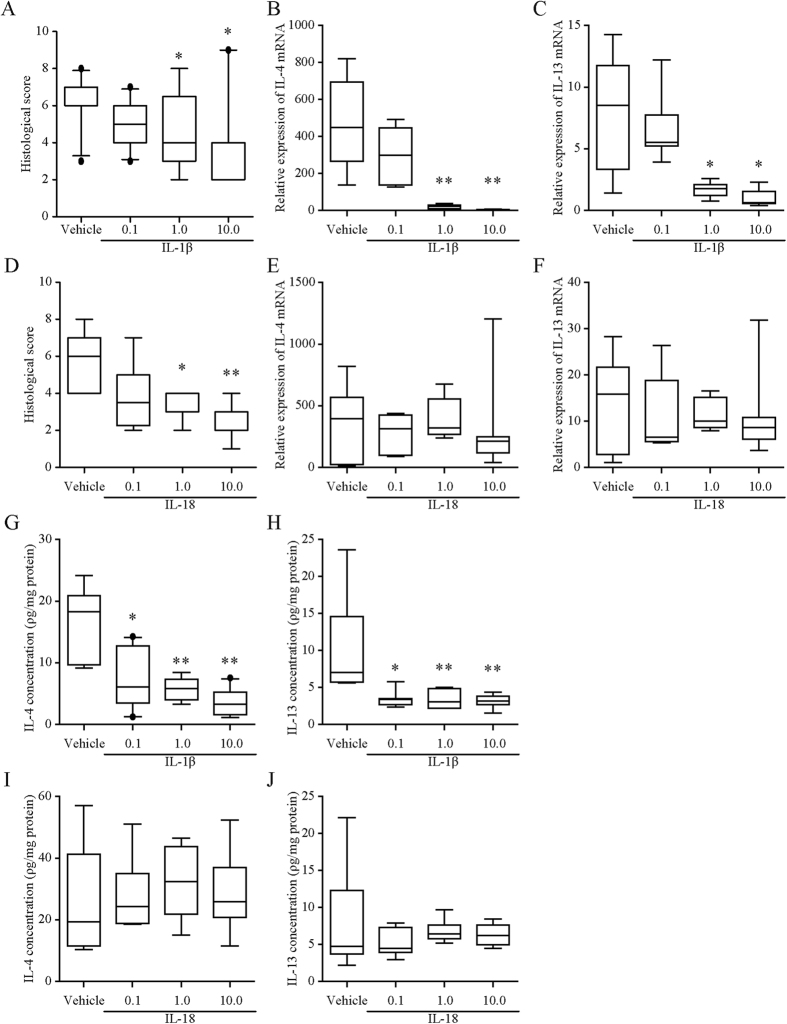
Effects of exogenous IL-1β and IL-18 on oxazolone-induced colitis. Mice received intraperitoneal injections of IL-1β (0.1–10 μg/kg), IL-18 (0.1–10 μg/kg), or vehicle after colitis induction. (**A**,**D**) Colonic histological score in mice given IL-1β (**A**) or IL-18 (**D**). Histological scores were calculated according to the criteria (Supplementary Table 4). **B**,**C**,**E**, and **F**: mRNA levels of IL-4 (**B**,**E**) and IL-13 (**C**,**F**) in oxazolone-induced mice colonic tissue after IL-1β (**B**,**C**) and IL-18 (**E**,**F**) administration. The levels of indicated mRNA were determined by quantitative reverse transcription-polymerase chain reaction. mRNA levels are expressed as ratios, relative to the mean value for normal colonic tissue. **G**–**J**: Protein concentrations of IL-4 (**G**,**I**) and IL-13 (**H**,**J**) in mice given IL-1β (**G**,**H**) or IL-18 (**I**,**J**) into OXA-treated mice. Protein concentrations were measured by the corresponding enzyme-linked immunosorbent assay. The protein levels in each sample were standardized using the modified bicinchoninic acid method. Each box plot represents median, 10–90% range, minimum, and maximum values. p values were calculated by post hoc steel significant difference multiple comparison. N = 7–10. **p < 0.01, *p < 0.05 compared with normal colonic tissue. O.D., optical density.

Exogenous administration of IL-1β at doses of 1 or 10 μg/kg immediately after and 24 h after rectal administration of OXA prevented the development of OXA-induced colitis in the mice (Fig. 5A), with a reduction in mRNA levels and protein concentrations of IL-4 and IL-13 (Fig. 5B,C,G,H) on day 1. In contrast, exogenous administration of IL-18 at doses of 1 or 10 μg/kg immediately after and 24 h after rectal administration of OXA also prevented the development of colitis (Fig. 5D) without affecting the expression of IL-4 and IL-13 (Fig. 5E,F,I,J) on day 1. Both IL-1β and IL-18 at a dose of 0.1 μg/kg had no preventive effect on colitis on day 1 (Fig. 5A,D).

### Genetic deletion of NLR3 and caspase-1 aggravated murine OXA-induced colitis

**Figure 6:**
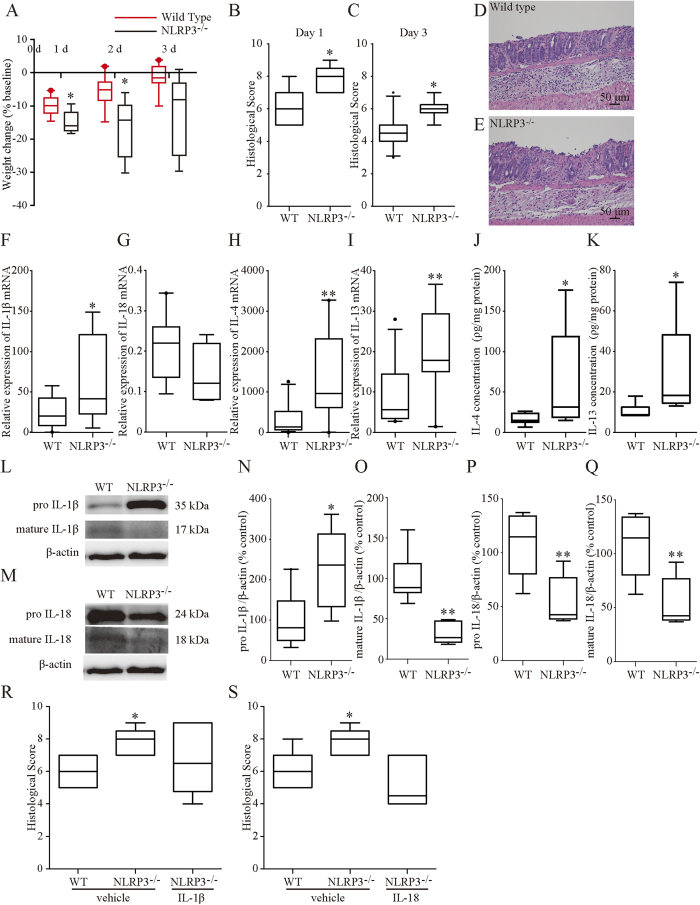
Effects of NLRP3 deficiency on oxazolone-induced colitis. (**A**) Changes in body weight during the experimental period. Round represents WT, wild-type. (**B**,**C**) Changes in histological score during experimental period. Histological scores were calculated according to the criteria (Supplementary Table 4). (**D**,**E**) Histological findings in wild-type (**D**) and NLRP3^−/−^ mice (**E**) with colitis on day 1. Compared to wild-type mice, NLRP3^−/−^ mice exhibited severe colitis on day 1. **F**–**I**: mRNA expression of IL-1β, IL-18, IL-4, and IL-13. The levels of indicated mRNA were determined by quantitative reverse transcription-polymerase chain reaction. mRNA levels are expressed as ratios, relative to the mean value for normal colonic tissue. (**J**,**K**) Protein concentrations of IL-4 and IL-13. Protein concentrations were measured by the corresponding enzyme-linked immunosorbent assay. The protein levels in each sample were standardized using the modified bicinchoninic acid method. (**L**–**Q**) Protein expression of IL-1β and IL-18. Pro- and mature IL-1β and pro- and mature IL-18 were investigated by western blot using its corresponding antibodies (Supplementary Table 2) and relevant bands were quantified with laser-scanning densitometry. β-actin was used as the normalization control. R, S: Changes in the histological score in mice given IL-1β or IL-18. NLRP3^−/−^ mice received intraperitoneal injections of IL-1β (0.1 μg/kg), IL-18 (0.1 μg/kg), or vehicle after colitis induction. Histological scores were calculated according to the criteria (Supplementary Table 4). Each box plot represents median, 10–90% range, minimum, and maximum values. p values were calculated by post hoc steel significant difference multiple comparison. N = 5–10. **p < 0.01, *p < 0.05 compared with wild type OXA-induced colitis tissue.

Colitis was induced by OXA treatment in NLRP3^−/−^ mice and caspase-1^−/−^ mice to determine the involvement of NLRP3 and caspase-1 in this disease process. NLRP3^−/−^ mice with colitis exhibited significantly greater weight loss than WT mice on days 1 and 2, and non-significantly greater weight loss than WT mice on day 3 (p = 0.053, Fig. 6A). Caspase-1^−/−^ mice with colitis exhibited significantly greater weight loss than WT mice on days 1, 2, and 3 (Supplementary Fig. 1A). The histological scores of both NLRP3^−/−^ and caspase-1^−/−^ mice were significantly higher than that of WT mice on day 1 and 3 (Fig. 6B–E, Supplementary Fig. 1B–E). As a control of whole-gut microbiota between WT and genetically deficient mice, we performed a part of these experiments using cohoused mice. Although these mice were older than the mice used in our other experiments and in other major studies because of the cohousing period, even after cohousing, similar results were obtained in terms of the histological findings and changes in body weights (Supplementary Fig. 2A–H). Therefore, we believe that the difference in gut microbes, if any, did not contribute to high sensitivity to OXA-induced colitis in NLRP3^−/−^ or caspase-1^−/−^ mice. Administration of either exogenous IL-1β or IL-18 at a dose of 0.1 μg/kg, which did not affect the severity of colitis in WT mice, immediately after and 24 h after rectal administration of OXA prevented progression of colitis in NLRP3^−/−^ mice as assessed by the histological score on day 1 of colitis induction (Fig. 6R,S). These findings revealed that the aggravation of pathology in NLRP3-deficient mice and caspase-1-deficient mice is due to deficiencies in mature IL-1β and IL-18.

The IL-1β mRNA and pro- IL-1β protein levels of NLRP3^−/−^ mice were higher compared to those of WT mice on day 1, whereas the mature IL-1β protein levels of NLRP3^−/−^ mice were significantly lower compared to those of WT mice (Fig. 6F,L,N,O). The IL-18 mRNA and both pro- and mature IL-18 protein levels of NLRP3^−/−^ mice were lower compared to WT mice (Fig. 6G,M,P,Q) on day 1. The IL-4 and IL-13 mRNA levels and protein concentrations of NLRP3^−/−^ mice were higher compared to those of WT mice (Fig. 6H–K) on day 1.

### Expression of mucin 2 (MUC2) in mice with OXA-induced colitis was induced by IL-18 administration

**Figure 7:**
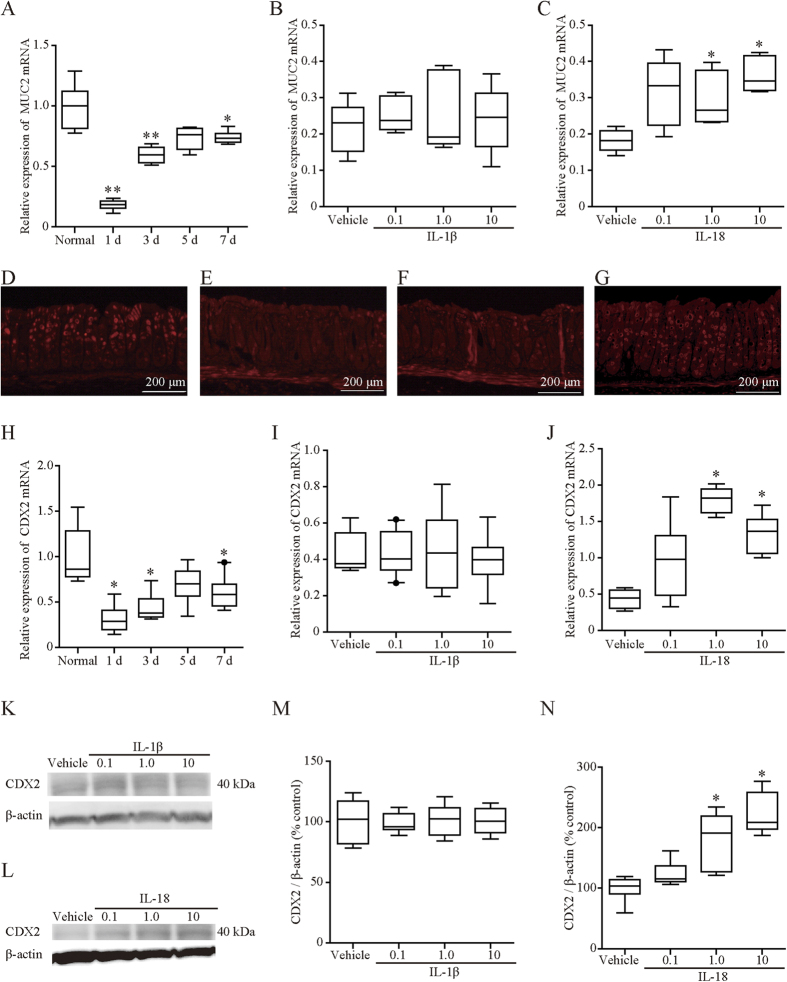
Expression of MUC2 and CDX2 during oxazolone-induced colitis. (**A**) MUC2 mRNA expression during oxazolone-induced colitis. (**B**,**C**) Effect of exogenous IL-1β (**B**) and IL-18 (**C**) on MUC2 mRNA expression. Mice received intraperitoneal injections of IL-1β (0.1–10 μg/kg) (**B**), IL-18 (0.1–10 μg/kg) (**C**), or vehicle after colitis induction. **D**–**G**: Immunohistochemical staining of MUC2. MUC2 was diffusely expressed on epithelial cells of the normal colon. (**D**) Colonic inflammation markedly reduced such expression (**E**), which was returned to the level of the normal colon by IL-18 (0.1 μg/kg) (**G**), but not IL-1β (0.1 μg/kg) (**F**). H: CDX2 mRNA expression during oxazolone-induced colitis. **I**,**J**: Effects of exogenous IL-1β or IL-18 on CDX2 mRNA expression. Mice received intraperitoneal injections of IL-1β (0.1–10 μg/kg) (**I**), IL-18 (0.1–10 μg/kg) (**J**), or vehicle after colitis induction. The levels of indicated mRNA were determined by quantitative reverse transcription-polymerase chain reaction. mRNA levels are expressed as ratios, relative to the mean value for normal colonic tissue. **K**–**N**: Protein expression of CDX2 one day after OXA administration. Expression levels of CDX2 (**K**–**N**) on day 1 were assessed by western blot analysis using the corresponding antibodies (Supplementary Table 2), and relevant bands were quantified by laser-scanning densitometry. β-Actin served as a normalization control. The mice received intraperitoneal injections of IL-1β (0.1–10 μg/kg) (**K**,**M**), IL-18 (0.1–10 μg/kg) (**L**,**N**), or vehicle after colitis induction. Protein levels are expressed as ratios, relative to the mean value for vehicle-treated colonic tissue. Each box plot represents median, 10–90% range, minimum, and maximum values. p values were calculated by post hoc steel significant difference multiple comparison. N = 5–14. **p < 0.01, *p < 0.05 compared with control colon tissue (**A**,**H**) or vehicle-treated colon tissue (**B**,**C**,**I**,**J**,**M**,**N**). The doses and sources of primary antibodies against MUC2 and CDX2 are listed in Supplementary Table 2.

MUC2 is the predominant intestinal mucin and forms an insoluble barrier that protects the intestinal epithelium against colonization by gut microbes^13^. Induction of colitis markedly reduced MUC2 mRNA expression on days 1, 3, and 7 (Fig. 7A). Exogenous administration of IL-18 at doses of 1 or 10 μg/kg reversed the reduction of MUC2 expression on day 1 (Fig. 7C), whereas IL-1β at any dose did not (Fig. 7B). Induction of colitis markedly reduced CDX2, which is a positive regulator of MUC2, mRNA expression on days 1, 3, and 7 (Fig. 7H). Both protein and mRNA expression levels of CDX2 were induced by exogenous administration of IL-18 at doses of 1 or 10 μg/kg on day 1 (Fig. 7J,L,N), whereas IL-1β at any dose did not (Fig. 7I,K,M).

Immunohistochemically, MUC2 was diffusely expressed on epithelial cells of the normal colon (Fig. 7D). Colonic inflammation markedly reduced the MUC2 expression pattern (Fig. 7E) on day 1, which was returned to the level of the normal colon by IL-18 (Fig. 7G), but not IL-1β (Fig. 7F).

## Discussion

Although several animal studies demonstrated that the NLRP3 inflammasome is involved in the pathogenesis of colitis, few studies exist that assess the role of the inflammasome in IBDs in humans. We demonstrated that in patients with UC, expression of the NLRP3 protein, whose large proportion was colocalized with cleaved caspase-1, was markedly enhanced in the inflamed colonic mucosa; expression levels of NLRP3 and colocalization of NLRP3 with cleaved caspase-1 positively correlated with disease severity. Although previous studies strongly suggested that the NLRP3 inflammasome plays a protective role in CD, experimental studies demonstrated that the NLRP3 inflammasome had two opposite effects on inflammation depending on the varying types of tissue injuries and inflammation^7^,^8^,^9^,^10^,^11^,^14^. Therefore, the result that the NLRP3 inflammasome was activated in UC leads to two possibilities. First, the inflammasome enhanced inflammation, resulting in aggravation of colonic damage. Second, in response to inflammation, the inflammasome was induced to resolve colitis and prevent further damage. Thus, we studied the role of the NLRP3 inflammasome by using an OXA-induced colitis model, a mouse UC model that is mediated by Th2 cytokines and showed the NLRP3 inflammasome has protective effects on OXA-induced colitis. Furthermore, NLRP3 expression in mice with OXA-induced colitis reached a peak by day1 when severity of the colitis was at a maximum. In addition, we observed a positive correlation of the percentage of NLRP3 and cleaved caspase-1 double-stained cells with the severity of colitis (Matts’ grading score) in patients with UC. These results strongly suggested that the NLRP3 inflammasome might play a protective role in the pathogenesis of UC.

Our data are consistent with several studies that demonstrated the NLRP3 inflammasome plays an important role in the maintenance of gut homeostasis^15^,^16^. Of note in these studies, DSS-induced colitis and/or TNBS-induced colitis were used to investigate the roles of the inflammasome. DSS-induced colitis is caused mainly by chemical stimulation and damage occurs with little immune system association, while TNBS-induced colitis is widely known as Th1 cell-mediated colitis. Thus, these models of colitis are inadequate to examine the pathogenesis of UC. Although recent studies showed the involvement of Th17 cytokines in the pathogenesis of UC as well as CD, these studies did not rule out the importance of Th2 cytokines in UC^17^,^18^. Thus, evaluation of the roles of Th2 cytokines in rodent models of colitis such as OXA-induced colitis should advance the understanding of the pathogenesis of UC. In this study, we used a mouse model of OXA-induced colitis, which is a well-known type of Th2-cell-mediated colitis, and this model is relevant to UC pathogenesis. To our knowledge, this is the first report on the role of the NLRP3 inflammasome in murine OXA-induced colitis.

Although we found that both IL-1β and IL-18 played a protective role in OXA-induced colitis, the roles of these cytokines on colonic inflammation are inconclusive and controversial. Previous studies have shown that IL-1β and IL-18 contribute to an enhancement of intestinal inflammation, resulting in aggravation of the damage^19^,^20^,^21^. However, several recent studies demonstrated that IL-18 derived from the NLRP3 inflammasome suppresses experimental colitis. Takagi *et al*. demonstrated that IL-18^−/−^ and IL-18 receptor^−/−^ mice were hypersusceptible to DSS-induced colitis, which was associated with higher mortality rates and more severe histopathological changes in these mice^22^. Dupaul-Chicoine *et al*. demonstrated that exogenous IL-18 administration rescues caspase-1^−/−^ mice from DSS-induced colitis with an improvement in the body weight loss and histopathological changes in these mice^9^. In contrast to IL-18, less attention has been focused on the role of IL-1β in colitis. We showed that exogenous IL-1β, as well as exogenous IL-18, improved OXA-induced colitis in WT mice and prevented worsening of colitis in NLRP3^−/−^ mice, strongly suggesting that the NLRP3 inflammasome-derived IL-1β may have an inhibitory effect on OXA-induced colitis. Thus, for a better understanding of the mechanisms involved in the NLRP3 inflammasome-associated inflammation and tissue damage, it seems to be insufficient to investigate the role of either one of the two cytokines independently of the other.

Of note, the dynamics strikingly differed between IL-1β and IL-18 during the development of OXA-induced colitis, whereas exogenous administration of either cytokine had a protective effect against the colitis. In response to induction of colitis, expression of IL-1β increased. Because exogenous IL-1β ameliorated OXA-induced colitis, this increase in IL-1β expression may be insufficient for prevention of overexpression of Th2 cytokines. On the other hand, IL-18 expression was diminished by the induction of colitis, while similarly to IL-1β, it is upregulated in several inflammatory conditions where the NLRP3 inflammasome is activated^10^. Although detailed mechanism behind the downregulation of IL-18 remains unclear, the dynamics of IL-18 expression may differ among different inflammatory conditions, and this downregulation may lead to enhancement of inflammation in OXA-induced colitis.

The effects of IL-1β and IL-18 on Th1/Th2 cytokine balance are controversial. Although IL-1 was originally reported to promote proliferation of Th2 clones, Shibuya *et al*. reported the requirement of IL-1 for IL-12-driven development of Th1^23^. IL-18 is considered to play a major role in the Th1 response^24^. However, several studies showed a positive role for IL-18 in promoting Th2 responses. During Leishmania species infection, IL-18 enhanced Th2 responses and susceptibility to this organism^25^. This cytokine has also been shown to elicit Th2 cytokines from mouse basophils^26^. Thus, the roles of IL-1β and IL-18 on Th1/Th2 cytokine balance may vary under different inflammatory conditions. Similarly, the role of NLRP3 in such cytokine balance is a topic of active debate. The NLRP3 inflammasome triggered Th2-biased inflammatory responses in asthma and atopic dermatitis^27^,^28^. On the contrary, several studies demonstrated stimulatory roles for this inflammasome in Th1-mediated inflammation and tissue injuries. NLRP3^−/−^ mice displayed less severe autoimmune encephalomyelitis with reduced IFN-γ production^29^, and this led to suppressed Th1 cell differentiation in a type 1 diabetic mouse model^30^. In this study, exogenous IL-1β reduced expression of colonic Th2 cytokines (IL-4 and IL-13) and improved OXA-induced colitis, while exogenous IL-18 also reduced severity of the colitis but without affecting expression of Th2 cytokines. In addition, NLRP3^−/−^ mice revealed severe colitis with increased expression of Th2 cytokines. This suggested that NLRP3-derived IL-1β may suppress Th2 cytokine production, leading to mitigation of the colonic damage and that IL-1β and IL-18 reduced the damage through different mechanisms.

MUC2, which is the predominant intestinal mucin, provides an insoluble barrier that serves to protect the intestinal epithelium. The findings that MUC2-deficient mice spontaneously develop colitis^31^,^32^ proved the protective effects of MUC2. Although the precise mechanism of regulation of MUC2 is still unclear, several inflammatory mediators such as inflammatory cytokines have been reported to modulate MUC2 expression. Iwashita *et al*. demonstrated that IL-4, IL-13, and TNF-α stimulated MUC2 mRNA expression through a mitogen-activated protein kinase pathway^33^. Caudal-related homeobox transcription factor 2 (CDX-2), which is a homeobox gene that encodes an intestine-specific transcription factor, is also known as a major regulator of MUC2 expression in the gastrointestinal tract. It is noteworthy that in this study, exogenous administration of IL-18 enhanced colonic MUC2 expression without affecting expression of IL-4 and IL-13 while upregulating CDX-2. Furthermore, a recent study reported that activation of the NLRP3/caspase-1/IL-18 axis induced MUC2 gene expression, resulting in an amelioration of DSS-induced colitis, which is consistent with our results^34^. Although other reports have shown that the expression of MUC2 is due to upregulation of IL-1β in *Shigella*-infected cells^35^ and in colorectal cancer cells^36^, both MUC2 expression and CDX-2 expression were not affected by IL-1β administration in our model of OXA-induced colitis. Thus, although the mechanism of MUC2 induction by IL-18 remains unknown, this stimulatory effect of IL-18 on MUC2 expression via CDX-2 upregulation seems to contribute to alleviation of colitis.

There are a few limitations of this study. First, the WT, NLRP3^−/−^, and Caspase-1^−/−^ mice came from different sources and have somewhat different genetic backgrounds. Colonic microflora of offspring is influenced by that of the dam. Differences in the microflora among the NLRP3^−/−^, caspase-1^−/−^, and C57BL/6 J mice may influence the results. Nonetheless, the phenotype of these knockout mice did not change after cohousing of these mice with WT mice, suggesting that the differences in gut microbes, if any, did not contribute to the high sensitivity to OXA-induced colitis in NLRP3^−/−^ or caspase-1^−/−^ mice. Second, we used a single model of Th2-mediated colitis, but currently, to the best of our knowledge, no other validated models of Th2-mediated colitis have been reported. Replication of our results on other models should strengthen our conclusions.

In conclusion, these results suggest that the NLRP3 inflammasome may have a protective effect on Th2 cytokines- and immune system-mediated experimental colitis; both the inflammasome derived IL-1β and IL-18 contribute to this protection via different mechanisms. Thus, the NLRP3 inflammasome may be a new therapeutic candidate for UC.

## Materials and Methods

### Human samples

Colonic tissue samples from operation resection specimens were obtained from 55 consecutive patients with UC (mean age: 45.7 years; age range: 20–85 years; male: 35 and female: 20) and seven control subjects (mean age: 70.8 years, age range: 53–87; male: 5 and female: 2). Our investigation was conducted according to the Declaration of Helsinki principles. All the patients provided informed consent for the use of their colon as a human sample. The ethics committee of Osaka City University Hospital approved our study (protocol number 2286).

### Histological evaluation of human samples

Tissue samples were fixed in periodate-lysine-paraformaldehyde and embedded in Tissue-Tek OCT Compound (Sakura Finetek Japan, Tokyo, Japan). Serial 4-μm-thick cryostat sections were mounted on silanized slides (Dako, Tokyo, Japan). The tissue samples were stained with hematoxylin/eosin (Wako, Osaka, Japan) in order to histologically evaluate them according to the Matts’ histopathological grading system (Supplementary Table 1)^12^.

Next, we evaluated the immunoreactivity of the human colon tissue. The primary antibodies, which were diluted by antibody diluent with background reducing components (Dako), were applied to the specimens and incubated overnight at 4 °C. The dosage and source of primary antibodies (NLRP3, cleaved caspase-1, and CD68) are listed in Supplementary Table 2. Then, the corresponding secondary fluorescent dye-conjugated antibodies (Alexa Fluor® 488 or 594; Invitrogen Corporation, Carlsbad, CA) were incubated with the specimens for 30 min at room temperature. Finally, DAPI Fluoromount-G™ (Southern Biotech, Birmingham, AL) was added to the sections. We then evaluated the sections using a fluorescent microscope equipped with argon and argon–krypton laser sources (Olympus, Tokyo, Japan). The NLRP3-positive cells or NLRP3 and cleaved caspase-1 double-stained cells among lamina propria cells were counted in three visual fields at 400× magnification in each tissue sample. Then, the average percentage of the NLRP3 positive cells relative to total cells were graded from 0 to 5. The detailed grading system is as follows: grade 0, none; grade 1, <20%; grade 2, 20–40%; grade 3, 40–60%; grade 4, 60–80%; grade 5, >80%.

### Animals

NLRP3^−/−^ and caspase-1^−/−^ mice on a C57BL/6 background were purchased from Jackson Laboratory (Bar Harbor, ME). WT C57BL/6 mice were purchased from Charles River Japan Inc. (Atsugi, Japan) and used as the control strain for these two knockout mouse strains. Specific pathogen-free (Supplemental Table 3) 5-week-old male animals were used. All the mice were housed in polycarbonate cages with paper chip bedding. The cages were located in an air-conditioned biohazard room with a 12-h light-dark cycle.

All experiments were carried out under the control of animal research committee in accordance with The Guidelines on Animal Experiments in Osaka City University Graduate School of Medicine, Japanese Government Animal Protection and Management Law (No. 105), and Japanese Government Notification on Feeding and Safekeeping of Animals (No. 6). All experimental procedures were approved by the Animal Care Committee of Osaka City University Graduate School of Medicine (Approval number 14031). We repeated the entire set of mice experiments at least twice on different days.

### Cohousing of mice

Some studies have shown that inflammasomes such as the NLRP3 and NLRP6 inflammasomes regulate colonic microbiota^10^,^37^,^38^. Therefore, to control gut microbiota, three WT mice were cohoused with three NLRP3^−/−^ or caspase-1^−/−^ mice for at least four weeks in order to minimize differences in gut microbiota, and then they were used in further experiments.

### Induction of OXA colitis

Colitis was induced by OXA (Sigma-Aldrich, St. Louis, MO) as previously described^2^. In brief, to pre-sensitize mice a 2 × 2 cm field of the abdominal skin was shaved and 200 μl of a 3% (w/v) solution of OXA in 100% ethanol (ETOH) (Wako) was applied. Five days later, 150 μl of 1% OXA in 50% ETOH or vehicle (ETOH alone) was administered intrarectally with an oral feeding needle for rats (Fuchigami, Kyoto, Japan) inserted 4 cm into the colon. After injection, mice were held in a vertical position for 30 s. The mice were euthanized 1, 3, 5, and 7 days after the intrarectal injection.

### Experimental groups

To evaluate the roles of IL-1β and IL-18 in the development of OXA-induced colitis in WT mice and NLRP3^−/−^ mice, the mice received intraperitoneal injections of mouse recombinant IL-1β (0.1–10 μg/kg; R&D Systems, Inc., Minneapolis, MN) or mouse recombinant IL-18 (0.1–10 μg/kg; Medical & Biological Laboratories Co., Ltd., Nagoya, Japan) or vehicle (saline only) immediately after and 24 h after rectal administration of ETOH or OXA. The mice were euthanized 4 h after the second intraperitoneal injection.

### Histological analysis in the mouse colon samples

Mouse colon samples were fixed in 10% buffered formalin phosphate. Samples were embedded in paraffin, and serial 4-μm sections were cut and mounted on silanized slides. The tissue samples were then stained with hematoxylin/eosin and graded from 0 to 15 using enterocyte loss, crypt inflammation, lamina propria mononuclear cells, neutrophils, and epithelial hyperplasia as criteria. (Supplementary Table 4)^39^.

For immunohistochemical staining, tissue samples were fixed in periodate-lysine-paraformaldehyde and embedded in Tissue-Tek OCT Compound. Serial 4-μm sections were cut and mounted on silanized slides. Expression levels of MUC2 were determined with an immunofluorescence method. The dosage and source of primary MUC2 antibodies are listed in Supplementary Table 2. The corresponding secondary fluorescent dye-conjugated antibodies (Alexa Fluor® 594; Invitrogen Corporation, Carlsbad, CA) were then incubated. As negative controls of immunofluorescence, tissue samples were incubated with PBS instead of the specific primary antibody and then reacted with the rabbit secondary antibodies. We confirmed that the rabbit secondary antibodies alone did not produce any fluorescent signals.

Finally, DAPI Fluoromount-G™ was added to the sections. We evaluated the sections using a fluorescent microscope equipped with argon and argon–krypton laser sources.

### RNA isolation and determination of the mRNA expression levels of inflammatory cytokines and inflammasome components in colon tissue using RT-PCR

Total RNA was isolated from colon tissue using an ISOGEN kit (Nippon Gene Co., Ltd., Tokyo, Japan) according to the manufacturer’s protocol. Complementary DNA was produced using the High Capacity RNA-to-cDNA Kit (Thermo Fisher Scientific Inc., Waltham, MA, USA) according to the manufacturer’s protocol. Real-time quantitative RT-PCR analyses were performed using an Applied Biosystems 7500 Fast Real-Time PCR system and software (Thermo Fisher Scientific Inc.). The reaction mixture was prepared according to the manufacturer’s protocol using the TaqMan Fast Universal PCR master mixture (Thermo Fisher Scientific Inc.). Thermal cycling conditions were as follows: 45 cycles of 95 °C for 15 s and 60 °C for 1 min. The expression levels of IL-1β, IL-18, TNF-α, NLRP3, caspase-1, MUC2, CDX2, IL-4, IL-13, and IFN-γ were quantified in colon tissue using real-time RT-PCR and standardized to TaqMan glyceraldehyde-3-phosphate dehydrogenase (GAPDH; Thermo Fisher Scientific Inc.) mRNA levels. The expression levels of these mRNAs are indicated as ratios to the mean value in normal colon tissue. The primers and probes used for RT-PCR are detailed in Supplementary Table 5.

### Western blotting

Colon tissues were homogenized and lysed on ice in a buffer containing 0.5% NP-40, 40 mM Tris-HCl (pH 8.0), 120 mM NaCl, phosphatase inhibitor cocktail (PhosSTOP, Roche Applied Science, Indianapolis, IN), and a protease cocktail inhibitor (Complete Mini, Thermo Fisher Scientific Inc., Waltham, MA). Protein levels in the lysate were measured with the modified bicinchoninic acid method (Thermo Fisher Scientific Inc.). Proteins were denatured with sodium dodecyl sulfate sample buffer at 95 °C for 5 min and then subjected to 15% sodium dodecyl sulfate-polyacrylamide gel electrophoresis and transferred to a PVDF membrane. Membranes were blocked in a Tris-buffered saline buffer (10 mM Tris-HCl, pH 7.5, 100 mM NaCl, 0.1% Tween-20) containing 5% skim milk and then incubated overnight with one of the antibodies (IL-1β, IL-18, caspase-1, NLRP3 and CDX2) (Supplementary Table 2). Bound antigen-antibody complexes were detected with the appropriate secondary antibodies coupled to HRP with enhanced chemiluminescence according to the manufacturer’s instructions (Amersham, Arlington Heights, IL). The membranes were stripped and re-probed with mouse anti-β-actin (Sigma-Aldrich Co.) as a loading control. Relevant bands were quantified with laser-scanning densitometry (Image Quant LAS 4000 mini, Image Quant TL Analysis Toolbox, GE Healthcare UK Ltd).

### Enzyme-linked immunosorbent assays for IL-4 and IL-13 in colon tissue

The amounts of IL-4 and IL-13 in the proteins from colon tissue were measured using mouse IL-4 Quantikine ELISA kit and mouse IL-13 Quantikine ELISA kit (R&D Systems Inc.) respectively, following the manufacturers’ instructions. The protein levels in each sample were standardized using the modified bicinchoninic acid method.

### Statistical analysis

For human studies, the correlation between the above score and Matts’ grade was analyzed using Spearman rank correlation coefficient for nonparametric data using IBM SPSS Statistics 19 (IBM Corporation, Armonk, NY).

All statistical tests for animal studies were performed using R software (version 3.2.2). Differences in mean values between groups were analyzed with the Mann-Whitney U test for the comparison of two groups while the Kruskal-Wallis test with steel post-hoc analysis was used for the comparison of more than three groups. A p value of <0.05 was considered significant.

## Additional Information

**How to cite this article**: Itani, S. *et al*. NLRP3 inflammasome has a protective effect against oxazolone-induced colitis: a possible role in ulcerative colitis. *Sci. Rep.* **6**, 39075; doi: 10.1038/srep39075 (2016).

**Publisher's note:** Springer Nature remains neutral with regard to jurisdictional claims in published maps and institutional affiliations.

## Supplementary Material

Supplementary Figure
