# Supplementary material for: NLRP3 inflammasome has a protective effect against oxazolone-induced colitis: a possible role in ulcerative colitis
Source: Sci Rep. 2016 Dec 14;6:39075. doi: 10.1038/srep39075 (PMC5155456; doi:10.1038/srep39075)
Supplement: Supplementary Figure [file srep39075-s1.pdf]

NLRP3 inflammasome has a protective effect against oxazolone-induced colitis: a possible role in ulcerative colitis

Shigehiro Itani, [1] MD, Toshio Watanabe, [1,2] MD, PhD, Yuji Nadatani, [1] MD, PhD, Naoki Sugimura, [1] MD, Sunao Shimada, [1] MD, Shogo Takeda, [1] MD, PhD, Koji Otani, [1] MD, PhD, Shuhei Hosomi, [1] MD, PhD, Yasuaki Nagami, [1] MD, PhD, Fumio Tanaka, [1] MD, PhD, Noriko Kamata, [1] MD, PhD, Hirokazu Yamagami, [1] MD, PhD, Tetsuya Tanigawa, [1,2] MD, PhD, Masatsugu Shiba, [1] MD, PhD, Kazunari Tominaga, [1,2] MD, PhD, Yasuhiro Fujiwara, [1,2] MD, PhD, Tetsuo Arakawa, [1] MD, PhD

[1]Department of Gastroenterology, Osaka City University Graduate School of Medicine, Osaka, Japan

[2]SAMURAI International GI Research Center, Osaka, Japan

Supplementary Figure 1. Effects of caspase-1 deficiency on oxazolone-induced colitis. A: Changes in body weight during the experimental period. Round represents WT, wild-type. B, C: Comparison of histological score between wild-type and caspase-1$^{-/-}$ mice on days 1 and 3. Histological scores were calculated according to the criteria (Supplementary Table 3). D, E: Histological findings in wild-type (D) and caspase-1$^{-/-}$ mice (E) with colitis. Compared to wild-type mice, caspase-1$^{-/-}$ mice exhibited severe colitis.

Supplementary Figure 2. The influence of cohousing on OXA-induced colitis in NLRP3$^{-/-}$ mice and caspase-1$^{-/-}$ mice. The negative effects of NLRP3 deficiency on body weights (A) and on the histological score of mice (B) were not attenuated by the cohousing of wild-type (WT) mice with NLRP3$^{-/-}$ mice. Histological findings in wild-type (C) and NLRP3$^{-/-}$ mice (D) with colitis. Compared to wild-type mice, NLRP3$^{-/-}$ mice exhibited severe colitis. The negative effects of caspase-1 deficiency on body weights (E) and on the histological score of mice (F) were not attenuated by the cohousing of wild-type mice with caspase-1$^{-/-}$ mice. Histological findings in wild-type (G) and caspase-1$^{-/-}$ mice (H) with colitis. Compared to wild-type mice, caspase-1$^{-/-}$ mice exhibited severe colitis.

Supplementary Figure 1.

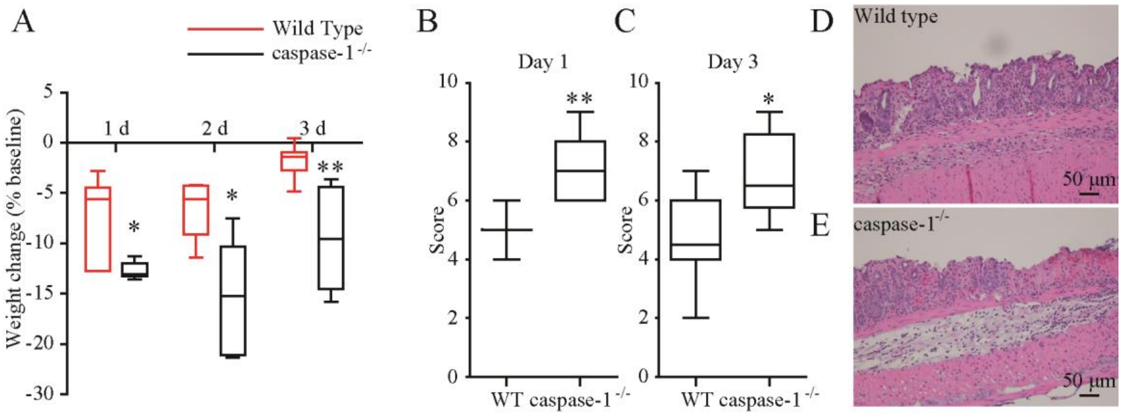

Supplementary Figure 2.

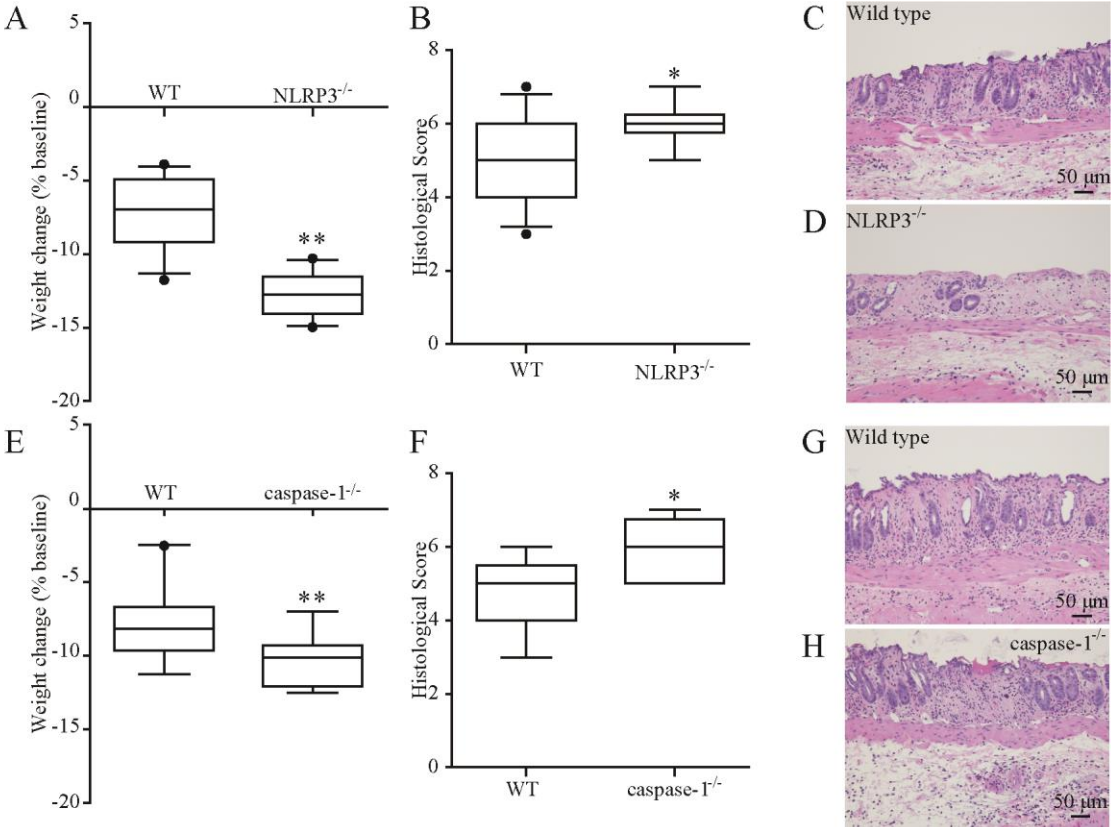

Supplementary Table 1. Matts' histopathological grading system

Grade 1	normal appearance
Grade 2	some infiltration of the mucosa or lamina propria with either round cells or polymorphs
Grade 3	much cellular infiltration of the mucosa, lamina propria, and submocosa
Grade 4	presence of crypt abcesses, with much infiltration of all layers of the mucosa
Grade 5	ulceration, erosion, or necrosis of the mucosa, with cellular infiltration of some or all of its layers

Supplementary Table 2. Antibodies Used

	Source information	Dosage	Use
NLRP3	mouse monoclonal (AdipoGen)	1:200 dilution	IHC (human)
cleaved caspase-1	rabbit polyclonal (EnoGene)	1:200 dilution	IHC (human)
CD68	rabbit polyclonal (LifeSpan BioSciences)	1:200 dilution	IHC (human)
CD68	mouse monoclonal (Abcam)	1:100 dilution	IHC (human)
MUC2	rabbit polyclonal (GeneTex)	1:800 dilution	IHC (mouse)
NLRP3	mouse monoclonal (AdipoGen)	1:1000 dilution	WB (mouse)
caspase-1	rabbit polyclonal (Santa Cruz)	1:200 dilution	WB (mouse)
IL-1β	goat polyclonal (R&D Systems)	1:500dilution	WB (mouse)
IL-18	rabbit polyclonal (Abnova)	1:500 dilution	WB (mouse)
CDX2	mouse monoclonal (Santa Cruz)	1:100 dilution	WB (mouse)
β-actin	mouse monoclonal (Sigma-Aldrich)	1:5000 dilution	WB (mouse)

Supplementary Table 3. Summary of checked specific pathogens.

		Charles River Laboratories International, Inc.	Laboratory Animal Center ,Graduate School of Medicine, Osaka City University
Bacteria			
	Pneumonia Virus of Mice	○	○
	Reovirus	○	
	Mouse Hepatitis Virus	○	○
	Murine Norovirus	○	
	Theiler's Murine Encephalomyelitis Virus	○	
	Minute Virus of Mice	○	
	Epizootic Diarrhea of Infant Mice Virus	○	
	Mouse Parvovirus (1 and 2)	○	
	Lymphocytic Choriomeningitis Virus	○	○
	Hantaan Virus	○	
	Mouse Adenovirus (1 and 2)	○	○
	Polyoma Virus	○	
	Mouse Pneumonitis Virus	○	
	Mouse Cytomegalovirus	○	
	Ectromelia virus	○	○
	Mouse Thymic Virus	○	
	Sendai Virus		○
Virus			
	Coryne bacterium kutscheri	○	○
	Pseudomonas aeruginosa	○	
	Salmonella spp.	○	○
	Streptococcus pneumoniae	○	
	Pasteurella pneumotropica	○	○
	Citrobacter rodentium	○	
	Helicobacter hepaticus	○	
	Helicobacter bilis	○	

	Helicobacter spp.	○	
	Streptobacillus moniliformis	○	
	Mycoplasma pulmonis	○	○
	Clostridium piliforme	○	○
	CAR Bacillus	○	
**Parasite, Fungus**			
	Ectoparasites	○	
	Gastrointestinal protozoa		
	-Spironucleus	○	○
	-Giardia	○	○
	-Non-Pathogenic Protozoa	○	
	Pinworms(Aspiculuris, Syphacia)	○	○
	Encephalitozoon cuniculi	○	

Supplementary Table 4. Histological injury score

Histological appearance		score
I	**Enterocyte loss**	
	Normal	0
	Loss of single cell	1
	Loss of groups of cells	2
	Frank ulceration	3
II	**Crypt inflammation**	
	Normal	0
	Single inflammatory cell	1
	Cryptitis	2
	Crypt abcess	3
III	**Lamina propria mononuclear cells**	
	Normal	0
	Slight increase	1
	Moderate increase	2
	Marked increase	3
IV	**Neutrophils**	
	Normal	0
	Slight increase	1
	Moderate increase	2
	Marked increase	3
V	**Epitherial hyperplasia**	
	Normal	0
	Mild	1
	Moderate	2
	Pseudopolyp	3

Supplementary Table 5. Primers and Probes

Gene		Primer and Probe
MUC2	Probe	5'-FAM-TCTGCATTGACTGGCGGAACCACACC-TAMRA-3'
	Primer (forward)	5'-ACCCTCTGTGCCAAGGAAGG-3'
	Primer (reverse)	5'-TGGGCACTTCACAGAGCAGA-3'
CDX2	Probe	5'-FAM-TTCCGCATCCACTCGCAGGTTTCG- TAMRA-3'
	Primer (forward)	5'-CCGCCGAACAGCTGTCCC-3'
	Primer (reverse)	5'-TCCTGGTTTTCACTTGGCTTCC-3'
TNF-α	Probe	5’-FAM-AATGGGCTTTCCGAATTCACTGGAGC-TAMRA-3’
	Primer (forward)	5’-TCATGCACCACCATCAAGGA-3’
	Primer (reverse)	5’-GAGGCAACCTGACCACTCTCC-3’
IL-1β	Probe	5’-FAM-ATGATCCCAATGAGTCGGCTGGAGA-TAMRA-3’
	Primer (forward)	5’-GCTGCTACTCATTCACTGGCAA-3’
	Primer (reverse)	5’-TGCTGCTGGTGATTCTCTTGTA-3’
IL-18	Probe	5’-FAM-TTCACAGAGAGGGTCACAGCCAGTCCT-TAMRA-3’
	Primer (forward)	5’-CCTGTGTTCGATATGACTGA-3’
	Primer (reverse)	5’-ACAGGAGAGGGTAGAGACATTTTACTA-3’
NLRP3	Probe	5’-FAM-AGGCCGGAATTCACCAACCCCAGCT-TAMRA-3’
	Primer (forward)	5’-TGCCTTGGGAGACTCAGGAG-3’
	Primer (reverse)	5’-CAGAGGTCAGAGCTGAACAACA-3’
caspase-1	Probe	5’-FAM-TGGCACATTTCCAGGACTGACTGGGACC-TAMRA-3’
	Primer (forward)	5’-GGACATCCTTCATTCATCCTCAGAAACA-3’
	Primer (reverse)	5’-TTTCTTTCCATAACTTCTGGGCTTT-3’
IL-4		Mm00445259_m1 (Thermo Fisher Scientific Inc.)
IL-13		Mm00434204_m1 (Thermo Fisher Scientific Inc.)
IFN-γ		Mm01168134_m1 (Thermo Fisher Scientific Inc.)
